# Advanced glycation end products promote the release of endothelial cell‐derived mitocytosis

**DOI:** 10.1002/2211-5463.70035

**Published:** 2025-04-08

**Authors:** Rong Liu, Yuhao Zhang, Tiantian Ge, Hao Wu, Yongbin Ma, Honghua Ye, Fei Guo

**Affiliations:** ^1^ Department of Cardiology The Affiliated Lihuili Hospital of Ningbo University China; ^2^ Ningbo Institute of Innovation for Combined Medicine and Engineering (NIIME), The Affiliated Lihuili Hospital of Ningbo University China; ^3^ The 1st Affiliated Hospital, Jiangxi Medical College, Nanchang University China; ^4^ Department of Central Laboratory Jintan Hospital, Jiangsu University Jintan China

**Keywords:** AGEs, diabetes, HUVECs, migrasomes, mitocytosis

## Abstract

Accumulation of advanced glycation end products (AGEs) and endothelial dysfunction are major factors that contribute to the progression of vascular complications in diabetes. Migrasomes, a newly discovered organelle involved in mitocytosis, play an important role in the selective removal of damaged mitochondria. Our research shows that human umbilical vein endothelial cells (HUVECs) can release migrasomes and undergo mitocytosis. In addition, when exposed to oxidative stress from AGEs, mitochondrial damage worsens, leading to the activation of migrasome‐mediated mitocytosis. We also found that migrasomes carrying mitochondria can be taken up by recipient cells. Understanding the connection between migrasome release, mitocytosis, and mitochondrial function in endothelial cells sheds light on the biological processes behind intercellular communication.

## Graphical abstract

Under diabetic conditions, AGEs induce mitochondrial damage in HUVECs, activating migrasome‐mediated mitocytosis. Migrasomes encapsulate damaged mitochondria and are released into the extracellular space, facilitating intercellular mitochondrial transfer. This study reveals a novel mechanism linking AGE‐driven mitochondrial dysfunction to migrasome‐dependent intercellular communication, offering therapeutic insights for diabetic vascular complications.

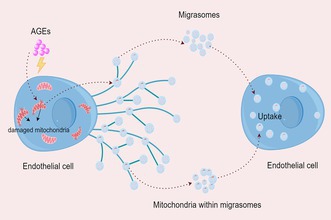

**Abbreviations**
AGEs: advanced glycation end products
AM: AGEs‐treated migrasomes
CM: control migrasomes
DM: diabetes mellitus
ECGS: endothelial cell growth supplement
ECL: enhanced chemiluminescence
ECM: endothelial cell medium
FBS: fetal bovine serum
HUVECs: human umbilical vein endothelial cells
Mito‐ROS: mitochondrial ROS
PBS: phosphate buffered saline
PVDF: polyvinylidene difluoride
ROS: reactive oxygen species
SDS/PAGE: sodium dodecyl sulfate/polyacrylamide gel electrophoresis
TEM: transmission electron microscopy
TSPAN4: tetraspanin 4
WGA: wheat germ agglutinin

Diabetes mellitus (DM) is currently a major public health problem, and vascular complications caused by DM are the main cause of death and disability in diabetic patients [1]. Hyperglycemia is a hallmark of diabetes and has been identified as one of the key factors in endothelial dysfunction [2]. On the one hand, hyperglycemia increases reactive oxygen species (ROS) in endothelial cells [3], which makes the cells unable to overcome oxidative stress. On the other hand, it also causes mitochondrial dysfunction and represents a crucial step in the development of endothelial dysfunction [4].

Persistent hyperglycemia leads to advanced glycation end products (AGEs) resulting from nonenzymatic glycation reactions with proteins, lipids, and nucleic acids, which is also one of the core pathophysiological factors of diabetic complications [5]. Many studies reported AGEs and its receptor RAGE activate numerous signaling pathways, leading to increased oxidative stress, inflammation, and mitochondrial dysfunction in endothelial cells [6, 7, 8].

The migrasomes are organelles rich in vesicle‐like structures formed in the contractile tail of migrating cells [9]. They have been reported to be involved in key cellular processes, including intercellular communication, mRNA and protein quality control, and mitochondrial quality control [10]. They can encapsulate a variety of contents, including mitochondria, and be swallowed by surrounding cells, providing a mechanism for material transfer between cells for cellular communication.

Mitochondria, as one of the most complex and important organelles, provide the necessary energy for cell activities. Strict mitochondrial quality control is necessary to maintain mitochondrial homeostasis [11]. Mitocytosis, a migrasome‐mediated mitochondrial quality control process, was proved by Professor Yu in 2021 and can couple mitochondrial homeostasis with cell migration [12]. Mitochondrial transfer between the cells is controlled by a variety of factors, including excreted organelles, intercellular mechanisms, and fused cells, so its functional outcomes are different [13].

AGEs are a diverse group of molecules produced through nonenzymatic glycosylation and are significant contributors to the development of diabetes and its complications [14]. Research indicates that AGEs can lead to oxidative stress [15] and disrupt mitochondrial quality control [16]. Specifically, AGEs have been shown to increase the production of ROS [17], which can overwhelm the cellular antioxidant defenses and lead to oxidative damage to cellular components, including lipids, proteins, and DNA [18]. Mitochondria are the main source of ROS and also the principal target of ROS attack. This oxidative stress is particularly detrimental to mitochondria, as it can impair mitochondrial function, damage mitochondrial DNA, and disrupt the mitochondrial membrane potential [19]. Consequently, AGE‐induced oxidative stress has been implicated in the disruption of mitochondrial quality control mechanisms, leading to the accumulation of damaged mitochondria within cells.

In our study, we discovered the release of migrasomes and the process of mitocytosis in endothelial cells. To study the changes in mitocytosis, the level of mitochondrial damage in endothelial cells was altered under AGE stimulation. Our research revealed that when endothelial cells experience injury, mitochondria are expelled through migration. We further explored that outgoing mitochondria can be transferred between cells through migrasomes. This work highlights the role of migrasomes and mitocytosis as important mediators in cellular communication, especially in relation to endothelial cell health and response to diabetic complications.

## Materials and methods

### Cell culture and treatment

Human umbilical vein endothelial cells (HUVECs) were purchased from Pricella (CP‐H082; Pricella, Wuhan, China). The cells were cultured in endothelial cell medium (#1001; ScienCell, https://sciencellonline.com/en/endothelial‐cell‐medium/) supplemented with endothelial cell growth supplement (ECGS), antibiotics (1% penicillin/streptomycin), and 5% fetal bovine serum (FBS). HUVECs were maintained at 37 °C in a humidified environment with 5% CO_2_. For subculturing, trypsin–EDTA was used. HUVECs were treated with or without 200 μg·mL^−1^ AGEs (bs‐1158P; Bioss, Beijing, China) or a control AGEs (bs‐1158PC; Bioss) for 24 h.

### Lentiviral infection of HUVECs

Lentiviral vectors expressing a pEX‐6 (pGCMV/MCS/RFP/Neo) against tetraspanin 4 (TSPAN4) were designed, constructed, amplified, and purified by GenePharma (Shanghai, China). HUVECs were seeded at a density of 1 × 10^4^ cells per well in 24‐well plates. The following day, cells were transiently transfected with 2.5 μg DNA using polybrene (G04011; GenePharma) according to the manufacturer's protocols and were subsequently incubated for 24 h.

### Reactive oxygen species detection

Reactive oxygen species levels were measured using DCFH‐DA probes (D6470; Solarbio, Beijing, China). HUVECs were seeded at a density of 2 × 10^5^ cells per well in 24‐well plates. After a 24‐h incubation with/without AGEs (200 μg·mL^−1^), the cell culture medium was discarded, and 500 μL of DCFH‐DA solution at a final concentration of 5 μm was added. The cells were then incubated at 37 °C for 20 min. Fluorescence intensity was observed using a fluorescence microscope (DMI6000B; Leica, Wetzlar, Germany). For semi‐quantitative evaluation, three regions from each group were selected, and the fluorescent signals were quantified using the imagej software (National Institutes of Health, Bethesda, MD, USA).

### Mitochondrial ROS detection

Mitochondrial ROS (Mito‐ROS) and superoxide were measured using the MitoSOX Red mitochondrial superoxide indicator (GC68230; GLPBIO, Montclair, CA, USA). Cultured HUVECs were seeded at a density of 2 × 10^5^ cells/well on 24‐well plates. The next day, cells were treated with or without AGEs (200 μg·mL^−1^) for 24 h. Then, the cell culture medium was discarded and 500 μL of Mito‐SOX Red solution at a final concentration of 5 μm was added. The cells were incubated at 37 °C for 20 min. A fluorescence microscope was used to observe the fluorescence intensity.

### Mitochondrial membrane potential (ΔΨm, MMP) detection

The JC‐1 assay kit (C2006; Beyotime, Shanghai, China) was used to measure the mitochondrial membrane potential in accordance with the manufacturer's guidelines, and nuclei were stained with Hoechst 33342 (C1029; Beyotime). Cultured HUVECs were seeded at a density of 2 × 10^5^ cells/well in 24‐well plates. On the next day, the cells were treated with/without AGEs (200 ng·mL^−1^) for 24 h. Then, the culture medium was discarded, and the cells were washed in phosphate buffered saline (PBS) once. The cells were incubated for 20 min with the JC‐1 staining solution at 37 °C, followed by two washes with the JC‐1 buffer solution. JC‐1 aggregates and monomers were visualized and captured using the fluorescence microscope.

### Migrasomes and mitochondrial tracker staining

Wheat Germ Agglutinin (WGA, 7528; Thermo Fisher, USA) and MitoTracker™Red (M7514; Thermo Fisher, Waltham, MA, USA) were used to visualize migrasomes and mitochondria, respectively. HUVECs were seeded at a density of 2 × 10^5^ cells per well on 35‐mm glass‐bottom dishes. The following day, cells were treated with PBS or AGEs (200 μg·mL^−1^) for 24 h. After discarding the medium, WGA and MitoTracker working solutions were added at final concentrations of 1 mm and 200 nm, respectively. After prewarming at 37 °C, the cells were incubated with the dyes for 15 min. The cells were then observed using a confocal microscope (DMI8; Leica).

### Isolation and characterization of migrasomes from HUVECs

HUVECs were inoculated in a 150‐mm dish (CLS430599; Corning, Corning, NY, USA) in endothelial cell medium (ECM) for 24 h. Cells and migrasomes on plates were washed twice with PBS, digested with 0.25% trypsin, and collected in 50‐mL tubes. Cell debris was removed by centrifugation at 1000 ***g*** for 10 min, followed by 4000 ***g*** for 20 min. The resultant supernatant was filtered through a 0.45‐μm filter, stopping immediately upon slight resistance. The filter was then reversed, and with the aid of a medical syringe containing 5 mL of PBS, the filtrate was collected and concentrated through a 100‐kDa ultrafiltration tube at 2000 ***g*** for 5 min at 4 °C, yielding pellets identified as migrasomes. Electron microscopy and western blotting were performed to assess the morphology and surface markers of the migrasomes.

### Transmission electron microscopy (TEM)

Migrasome morphology was examined using TEM. A 20 μL aliquot of the migrasome suspension was placed onto a copper grid with a carbon film for 3–5 min. Subsequently, 2% phosphotungstic acid was applied to stain for 1–2 min at room temperature. Images were captured using a Hitachi HT‐7800 transmission electron microscope (TEM) at 80 kV. Additionally, mitochondrial morphology and mitophagosomes from HUVECs were analyzed via TEM. Samples were fixed in 2.5% glutaraldehyde, postfixed in 2% osmium tetroxide, dehydrated through graded ethanol, and embedded in epoxy resin. Ultrathin sections (60–80 nm) were cut with a diamond knife, placed on Formvar‐coated copper grids (150 mesh), stained with 5% uranyl acetate for 15 min, followed by 0.1% lead citrate for 5 min.

### Western blot analysis

Migrasome lysates were quantified using a BCA Protein Assay Kit (P0012S; Beyotime). Equal amounts of protein were subjected to 10% sodium dodecyl sulfate/polyacrylamide gel electrophoresis (SDS/PAGE) (P0015A; Beyotime) and transferred to polyvinylidene difluoride (PVDF) membranes (FFP39; Beyotime). The membranes were blocked using 4% skimmed milk in TBST (G0004‐1L; Servicebio, Wuhan, China) and subsequently incubated with primary antibodies against TSPAN4 (A10253; ABclonal, Wuhan, China). Following this, the membranes were incubated with HRP‐conjugated secondary antibodies (RGAR001; Proteintech, Wuhan, China), and enhanced chemiluminescence (ECL) reagent (P10100; NCM Biotech, Suzhou, China) was used for detection.

### Analysis of cellular uptake of migrasomes

To assess cellular uptake of migrasomes, HUVECs were seeded at a density of 2 × 10^5^ cells·mL^−1^ and incubated with migrasomes at 37 °C for 2 h. Following this incubation, the cells were labeled with the DiO dye (ID5570; Solarbio) and Hoechst 33342 (C0031; Solarbio) according to the manufacturer's instructions. DiR (IC6110; Solarbio) and mitoTracker™Red were utilized to visualize migrasomes and mitochondrial content within the migrasomes, respectively.

In brief, HUVECs were stained with 5 μm DiO dye and 1 μg·mL^−1^ Hoechst for 20 min at 37 °C in the dark. Then, a solution of 5 μm DiR dye or MitoTracker™Red dye was added to the migrasome suspension and incubated for 30 min in the dark.

Next, the HUVECs and migrasomes were cocultured for 2 h at 37 °C in the dark (Fig. 3D). Fluorescence images (showing all red, green and blue) were captured using a confocal microscope.

### Statistical analysis

Statistical analyses were conducted using graphpad prism version 8.3.0 (GraphPad Software, Inc., La Jolla, CA, USA). Results from all experiments are expressed as mean ± standard error of the mean. The Student's *t*‐test was used to compare the two groups of data. Statistical significance was set at a *P* value of < 0.05 (*P < 0.05, **P < 0.01, ***P < 0.001). *P* > 0.05 was represented by “ns,” indicating no statistical significance.

## Results

### HUVEC can release migrasomes and mitocytosis

**Fig. 1:**
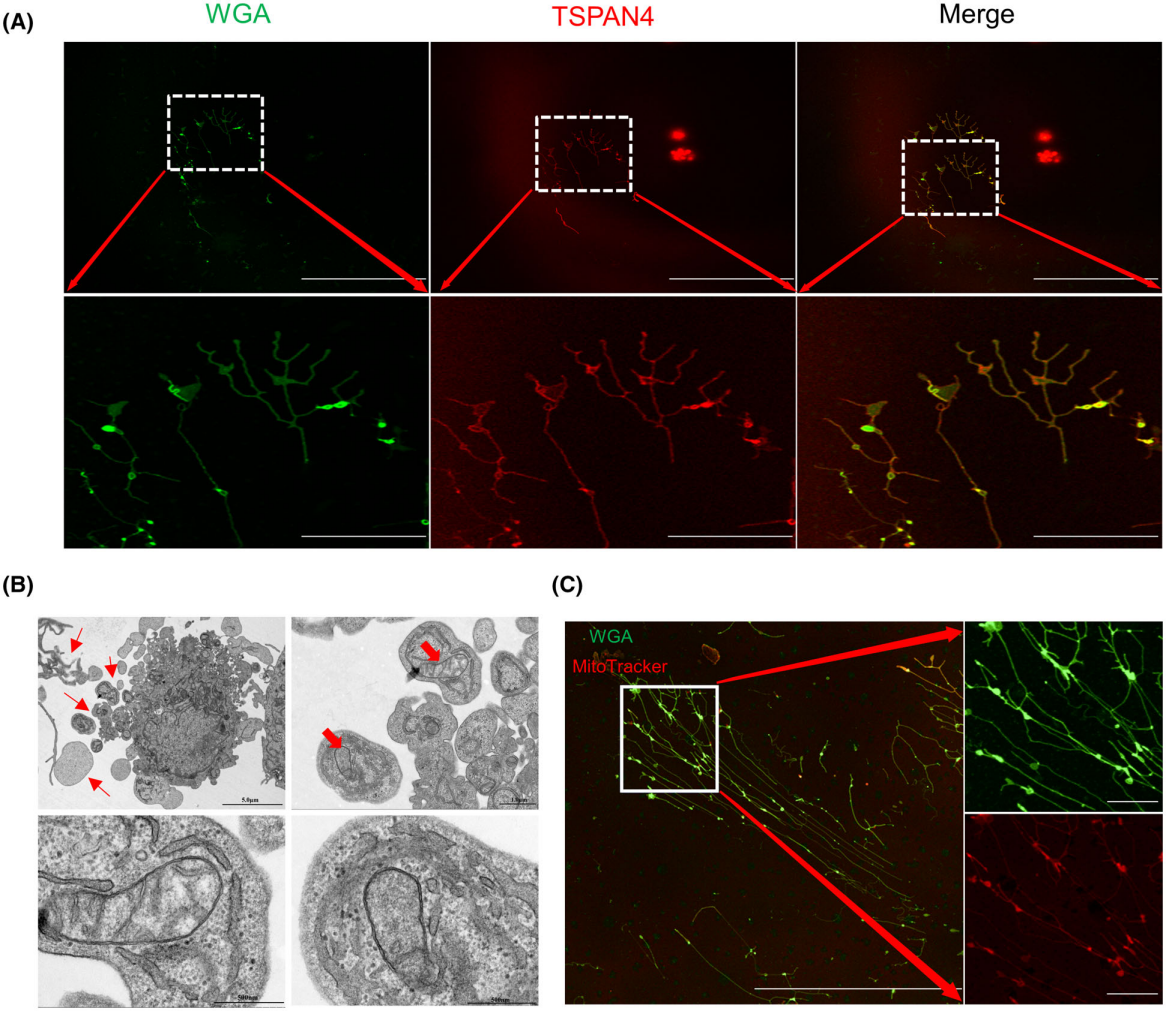
HUVECs release migrasomes and exhibit mitocytosis. (A) Colocalization of TSPAN4 with WGA in HUVECs. HUVECs were transfected with LV8N‐TSPAN4 and immunofluorescently labeled for Flag (red) and migrasomes (green), *n* = 3. Scale bar = 81.9 μm. The lower panels depict an enlarged ROI with a scale bar = 20 μm. (B) TEM reveals migrasomes and mitocytosis released by HUVECs, *n* = 3. In the upper panels, the left Scale bar = 5 μm and the right Scale bar = 1 μm. The lower panels depict an enlarged ROI with a scale bar = 500 nm. (C) Confocal image of HUVECS stained with WGA, MitoTracker, *n* = 3. Scale bar = 81.9 μm; The right panels, an enlarged ROI is shown with a scale bar of 20 μm. HUVECs, human umbilical vein endothelial cells; ROI, region of interest; TEM, transmission electron microscopy; TSPAN4, tetraspanin‐4; WGA, wheat‐germ agglutinin.

Recent study by Yu *et al*. [9] discovered that during the process of cell migration, “migrating cells” can secrete migrasomes, a unique class of microvesicles with a size between 500 and 3000 nm. Given that HUVECs have the capability to migrate, we hypothesize that HUVECs can also release migrasomes. To verify this hypothesis, we transfected HUVECs with lentivirus expressing TSPAN4‐RFP [20], and combined this with WGA staining [21] to jointly label the migrasomes. During *in vitro* culture, we closely monitored the movement trajectory of HUVECs, and observed TSPAN4‐RFP and WGA‐labeled migrasomes on culture dishes following cell migration. The release of migrasomes along the tubular structures confirmed that HUVECs can indeed release these microvesicles (Fig. 1A). To further verify our findings, we digested and collected the cells, then examined them using TEM. We clearly observed retraction fibers with tubular structures around the cells, from which vesicle‐like structures emerged at the tips and intersections (Fig. 1B). These structures exhibited varying numbers of vesicles and resembled the distinctive open pomegranate shape typical of migrasomes [9].

In addition to their role in secretion, migrasomes are involved in mitochondrial transport and expulsion, which are critical for maintaining mitochondrial homeostasis [22]. Impaired mitochondria are packaged and selectively removed through a process known as mitocytosis, functioning as a form of mitochondrial quality control [12]. Extracellular mitochondria, regardless of their structural or functional integrity, may play essential roles in intercellular communication and immune responses [23, 24, 25]. To capture the phenomenon of migrasome‐mediated mitocytosis, we stained HUVECs with wheat germ agglutinin (WGA) and MitoTracker. Confocal microscopy revealed the expulsion of mitochondria that were wrapped by migrasomes (Fig. 1C). Collectively, our findings illustrate the successful capture of both migrasome release and mitocytosis in HUVECs, highlighting the intricate relationship between these processes in cellular function.

### AGEs induce mitochondrial damage and migrasome‐mediated mitocytosis in HUVECs

**Fig. 2:**
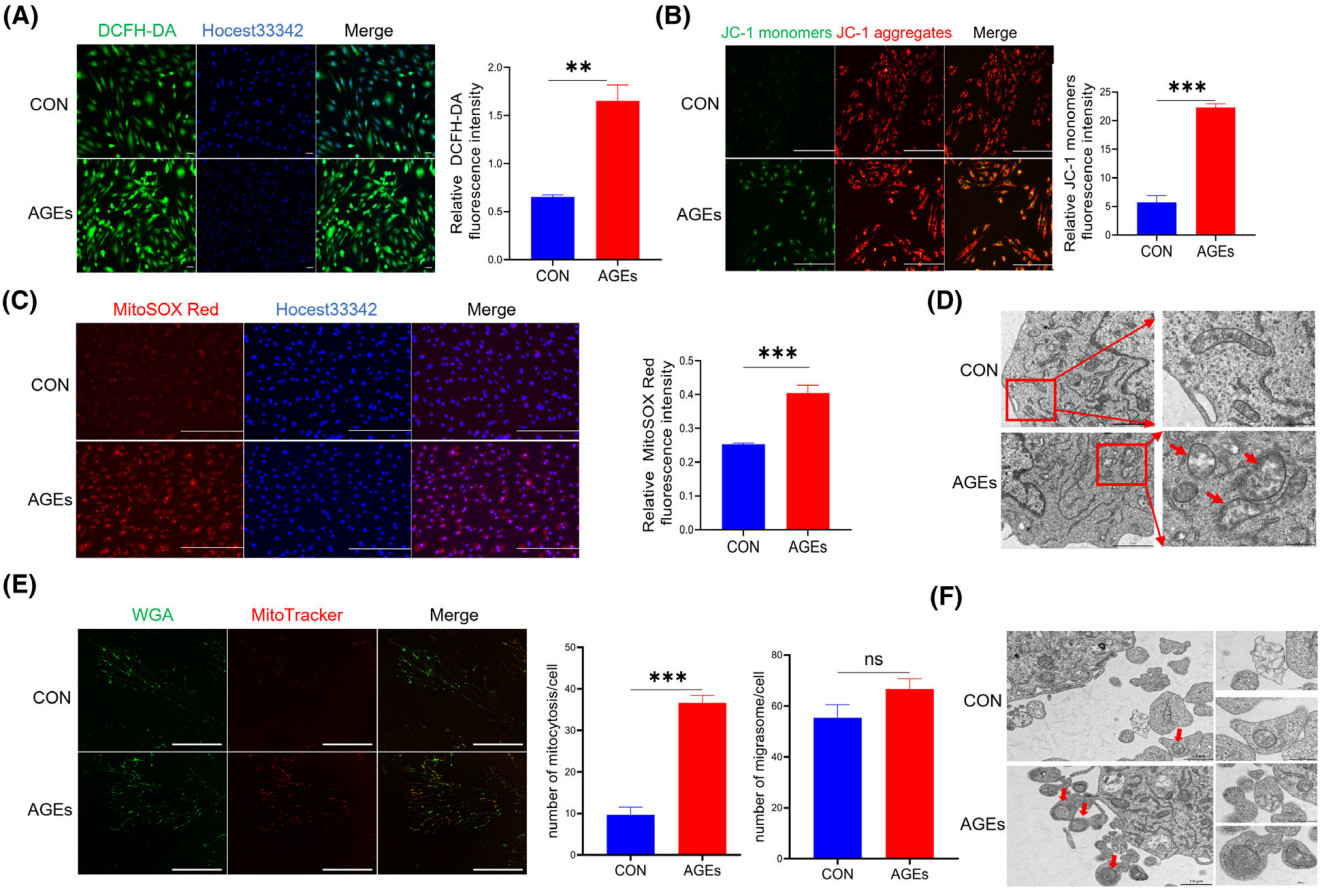
AGEs induce mitochondrial damage and promote migrasome‐mediated mitocytosis in HUVECs. (A) HIUVECs were treated with AGEs, leading to oxidative stress, *n* = 3/group. Scale bar = 70 μm. The graph on the right shows the relative fluorescence intensity of DCFH‐DA. (B) Mitochondrial membrane potential of HIUVECs was treated with AGEs, *n* = 3/group. Scale bar = 250 μm. The graph on the right shows the relative fluorescence intensity of JC‐1 monomers. (C) Mitochondrial oxidative stress of HIUVECs was treated with AGEs, *n* = 3/group. Scale bar = 250 μm. The graph on the right shows the relative fluorescence intensity of MitoSOX Red. (D) Representative TEM results show mitophagy in HUVECs incubated with AGEs or control AGEs for 24 h, *n* = 3/group. Red arrowheads indicate mitochondrial disruption and mitophagy‐like structures. Scale bar (left) = 2 μm; scale bar (right) = 500 nm. (E) Representative confocal image taken after HUVECs were treated with AGEs for 24 h, stained with WGA and MitoTracker, *n* = 3/group. Scale bar = 81.9 μm. The graphs on the right display the statistical count of migrasomes per cell and the number of mitocytosis events per cell. (F) TEM was used to observe migrasomes and mitochondria within the migrasomes, with red arrowheads highlighting migrasome‐mediated mitocytosis, *n* = 3/group. Scale bar = 1 μm; for the right panels, an enlarged ROI is shown with a scale bar of 500 nm. AGEs, advanced glycation end products; TEM, transmission electron microscopy; WGA, wheat‐germ agglutinin. Data in A–C and E are quantified with three random fields for each sample. The error bars represent the mean ± SEM (standard error of the mean) with the indicated significance. Statistical analysis utilized Student's *t*‐test (ns, no significance; *P* > 0.05, *P < 0.05, **P < 0.01, ***P < 0.001, and ****P < 0.0001).

Research has indicated that AGEs can lead to oxidative stress [15] and disrupt mitochondrial quality control [16]. To assess the effects of AGEs on mitochondrial oxidative stress and damage, we used the DCFH‐DA probe to measure changes in ROS. The results revealed a significant increase in ROS levels following AGE treatment (Fig. 2A). We conducted additional experiments using mitochondrial membrane potential (ΔΨm, MMP) detection and Mitochondrial ROS detection to further elucidate the mitochondrial dysfunction induced by AGE treatment. JC‐1 staining revealed a significant decrease in mitochondrial membrane potential (ΔΨm) in AGE‐treated HUVECs, as indicated by the increased green fluorescence compared with the red fluorescence, suggesting impaired mitochondrial function (Fig. 2B). Meanwhile, MitoSOX Red staining showed a marked increase in mitochondrial superoxide levels in AGE‐treated cells, evidenced by enhanced red fluorescence within mitochondria (Fig. 2C). These findings, combined with our results using DCFH‐DA (Fig. 2A) that demonstrated elevated overall cellular oxidative stress, collectively support the conclusion that AGEs treatment induces mitochondrial dysfunction and oxidative damage.

Mitophagy is essential for maintaining mitochondrial health by isolating damaged or depolarized mitochondria into autophagosomes for lysosomal degradation. We conducted a comparative analysis using electron microscopy to investigate the subcellular structure of HUVECs treated with and without AGEs. Our findings revealed abnormal mitochondrial morphology along with an obvious increased occurrence of mitophagy within the HUVECs obtained from AGEs (Fig. 2D).

To investigate migrasome‐mediated mitocytosis, HUVECs were treated with and without AGEs for 24 h. The release of mitochondria enclosed by migrasomes was observed and quantified using confocal microscopy with WGA and MitoTracker staining. The results indicate that HUVECs can generate migrasomes, and AGEs enhance mitocytosis (Fig. 2E). Importantly, migrasomes and retraction fibers in AGE‐treated HUVECs were more dispersed, and the extent of mitocytosis was significantly greater. Under TEM, we also noted increased mitocytosis and mitochondrial membrane defects in the migrasomes from the AGE‐treated group, suggesting that severely damaged mitochondria are selectively transported through mitocytosis (Fig. 2F).

In conclusion, the combined results from JC‐1, MitoSOX Red, DCFH‐DA, and TEM analyses provide robust evidence that AGE‐induced mitochondrial dysfunction is associated with enhanced mitocytosis and migrasome‐mediated mitochondrial expulsion. This highlights the critical role of migrasomes in maintaining mitochondrial quality control in response to mitochondrial dysfunction.

### Characterization of migrasomes

**Fig. 3:**
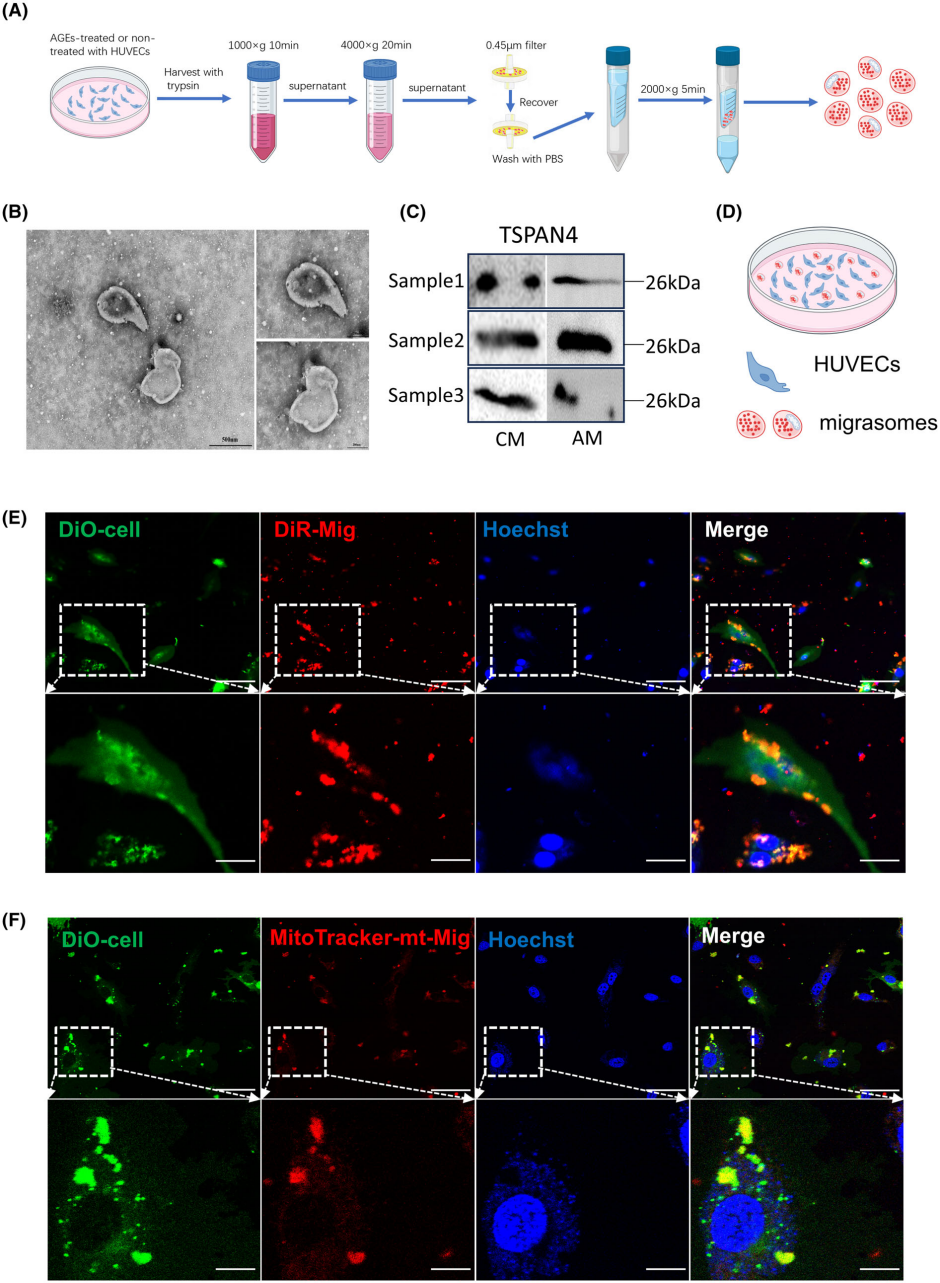
Characterization and uptake of migrasomes isolated from HUVECs. (A) Schematic representation of the isolation process for migrasomes derived from cultured HUVECs. (B) Electron microscopic picture of migrasomes, *n* = 3. Left image scale bar = 500 nm; Right image scale bar = 200 nm. (C) Western blot (WB) analyses confirm the presence of specific markers within the extracted migrasomes. Sample 1–3 represent three independent replicates of western blot analysis for both CM and AM groups. CM represents CON migrasomes; AM represents AGEs‐treated migrasomes. (D) Schematic illustration of co‐culture experiments involving HUVECs and migrasomes. (E) Coculture of migrasomes (Red) and HUVECs (Green), with images captured via confocal microscopy to observe the transfer of migrasomes (Mig) to HUVECs, *n* = 3. Scale bar = 10 μm. The lower panels depict an enlarged ROI with a scale bar = 2.5 μm. Fluorescent markers: Red—DiR; Green—DiO; Blue—Hoechst. (F) Confocal microscopy images illustrating the transfer of mitochondria within migrasomes (mt‐Mig) (Red) to HUVECs (Green), *n* = 3. Scale bar = 10 μm. The lower panels depict an enlarged ROI with a scale bar =2.5 μm. Fluorescent markers: Red—MitoTracker; Green—DiO; Blue—Hoechst.

Given that AGEs can enhance the release of migrasomes and promote mitocytosis, we isolated the migrasomes from the HUVECs. We implemented a novel protocol that enables quick isolation of these structures from the cultured cells (Fig. 3A) [26]. To further characterize their morphology, we utilized TEM, revealing that migrasomes are circular vesicles of varying sizes (Fig. 3B). Additionally, TSPAN4 is a well‐established marker for migrasomes [27, 28]. Therefore, we conducted western blot analysis, which confirmed the presence of migrasome structures in our isolated vesicles (Fig. 3C). In our study, control migrasomes (CM) refers to migrasomes isolated from untreated HUVECs, while AGE‐treated migrasomes (AM) refers to migrasomes isolated from HUVECs treated with AGEs. To ensure the reproducibility and reliability of our results, we performed western blot analysis on three independent replicates for each group (Samples 1–3). These replicates represent three separate isolation and analysis experiments for both CM and AM groups.

Overall, the combination of TEM imaging to visualize the morphological features of migrasomes and western blot analysis to detect the migrasome marker TSPAN4 demonstrates that we successfully isolated high‐purity migrasomes from HUVECs. These results, together with our other characterizations, provide a comprehensive validation of the migrasomes used in our study.

### Uptake of migrasomes and migrasome‐mediated mitocytosis

To further explore the migrasome‐mediated substance delivery between cells, we isolated migrasomes from HUVECs and cocultured them with HUVECs for a duration of 2 h (Fig. 3D). To observe this interaction directly, the cells were stained with DiO and Hoechst, while the migrasomes and the mitochondria within them were labeled with DIR and mitoTracker, respectively. Confocal microscopy revealed that migrasomes and mitochondria within migrasomes were internalized by HUVECs, with some migrasomes intersecting the cell membrane (Fig. 3E,F, Figs S1 and S2). These observations indicate that both migrasomes and the mitochondria they contain can be phagocytosed during coculture with HUVECs.

## Discussion

In this study, we explored the role of migrasomes in the process of mitocytosis within HUVECs, particularly focusing on the impact of oxidative stress induced by AGEs, which are linked to complications in diabetes. Our findings reveal that migrasomes can effectively transport mitochondrial components to recipient cells, underscoring their potential importance in intercellular communication and mitochondrial maintenance.

Vascular complications, a pressing concern for global health, significantly threaten human well‐being [29]. ECs line the innermost layer of blood vessels, playing a crucial role in sustaining vascular homeostasis by regulating various molecules. They possess a complex network of vesicles that contribute not only to the biogenesis of extracellular vesicles but also to the exchange of macromolecules between the bloodstream and endothelial cells.

Migrasomes, identified as novel organelles ranging from 0.5 to 3 μm in diameter, have recently gained attention for their diverse roles across various cell types, tissues, organs, and body fluids [9]. These organelles contain a wide array of substances relevant to both normal physiological processes and numerous diseases, including both benign and malignant conditions [30]. Our study not only confirms the release of migrasomes by HUVECs during cell migration using advanced imaging techniques (confocal microscopy and TEM) but also reveals the presence of damaged mitochondria within these migrasomes. This finding is significant as it provides novel insights into the role of migrasomes in cellular processes and their potential involvement in intercellular communication and waste disposal.

Additionally, our research indicates that AGEs are associated with elevated ROS levels, causing mitochondrial damage and enhancing migrasome‐mediated mitocytosis. We confirmed these effects through experiments detecting mitochondrial membrane potential and oxidative stress levels. The results show that AGEs significantly depolarize mitochondrial membrane potential and increase ROS levels within mitochondria, leading to impaired mitochondrial function. The findings suggest that this process is linked to impaired mitochondrial quality control, with AGEs promoting the expulsion of damaged mitochondria wrapped in migrasomes. Overall, the study underscores the relationship between intracellular mitochondrial dysfunction and migrasome dynamics in HUVECs.

Migrasomes play an active role in maintaining mitochondrial health within endothelial cells, rather than being mere byproducts of cell migration. Mitocytosis facilitates migrasome‐mediated mitochondrial transfer, helping mitigate mitochondrial stress within cells [12]. The selective expulsion of dysfunctional mitochondria through mitocytosis promotes cellular quality control, crucial for maintaining mitochondrial function in the face of oxidative stress. By effectively removing impaired mitochondria, this process helps prevent cellular dysfunction and supports overall cellular health.

Furthermore, these vesicles influence vascular health, particularly concerning diabetes‐associated conditions, by mediating intercellular communication and potentially mitigating damage to endothelial function. Our findings indicate that AGEs lead to significant abnormalities in mitochondrial morphology, which promote increased mitophagy, highlighting that chronic exposure to AGEs disrupts mitochondrial quality control. In response to mitochondrial damage and stress, mitocytosis emerges as a protective cellular strategy, allowing for the removal of impaired mitochondria to preserve cellular health.

Endothelial extracellular vesicles act as mediators of intercellular communication, transmitting biological information from donor cells to recipient cells and exerting both harmful and beneficial effects on vascular function [31]. The differing biogenesis and release of extracellular vesicles containing various cargoes significantly affect the biological processes in recipient endothelial cells, thereby regulating endothelial homeostasis [32]. Although migrasomes and exosomes are both extracellular membrane‐bound vesicular structures, a comparison of their proteomics revealed that these two structures share only 27% of proteins and exhibit significant differences in classical membrane markers, specific protein markers, and release processes [33]. It has been shown that extracellular vesicles derived from AGE‐stimulated HUVECs protect against diabetes‐associated vascular calcification, suggesting a protective role [34]. Recent studies propose that intercellular mitochondrial transfer may also play a crucial role in regulating mitochondrial function within recipient cells [35]. This research highlights that migrasomes can carry selected mitochondrial cargoes to target cells, in response to internal and external cues.

In summary, our findings illuminate the intricate interplay between migrasome release, mitocytosis, and mitochondrial homeostasis in ECs, particularly in the context of oxidative stress induced by AGEs. This research enhances the understanding of endothelial cell biology and paves the way for potential therapeutic strategies aimed at mitigating mitochondrial dysfunction associated with chronic diseases such as diabetes. Future research should explore the signaling pathways involved in these processes, as well as the implications of migrasome‐mediated mitocytosis in other cell types and disease states.

## Conflict of interest

The authors declare no conflict of interest.

## Author contributions

RL was involved in conceptualization, formal analysis, investigation, methodology, visualization, and writing original draft. YZ was involved in formal analysis, investigation, methodology, and visualization. TG was involved in formal analysis and writing original draft. HW was involved in investigation, methodology, and visualization. YM was involved in conceptualization, methodology, and writing review & editing. HY was involved in funding acquisition and writing review & editing. FG was involved in conceptualization, funding acquisition, project administration supervision, and writing review & editing. All authors approved the final version of the manuscript.

## Supporting information


**Fig. S1.** 3D view of the internalization of migrasomes by human umbilical vein endothelial cells.
**Fig. S2.** 3D view of the transfer of mitochondria within migrasomes to human umbilical vein endothelial cells.

## Data Availability

All data were available from the corresponding author upon reasonable request.
