# Supplementary material for: Advanced glycation end products promote the release of endothelial cell‐derived mitocytosis
Source: FEBS Open Bio. 2025 Apr 8;15(7):1068–78. doi: 10.1002/2211-5463.70035 (PMC12226421; doi:10.1002/2211-5463.70035)
Supplement: Supplementary file 1 — Fig. S1. 3D view of the internalization of migrasomes by human umbilical vein endothelial cells. Fig. S2. 3D view of the transfer of mitochondria within migrasomes to human umbilical vein endothelial cells. [file FEB4-15-1068-s001.docx]

Supplementary Figures


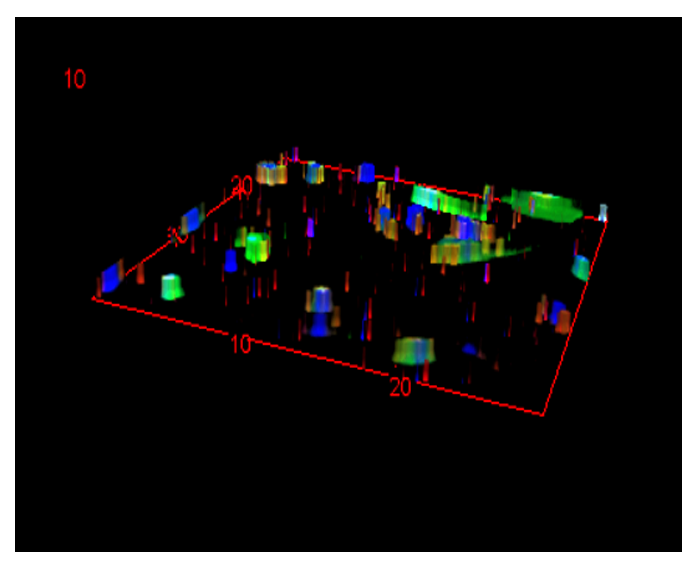


Supplementary Figure S1 3D view of the internalization of migrasomes(Red) by HUVECs (Green).


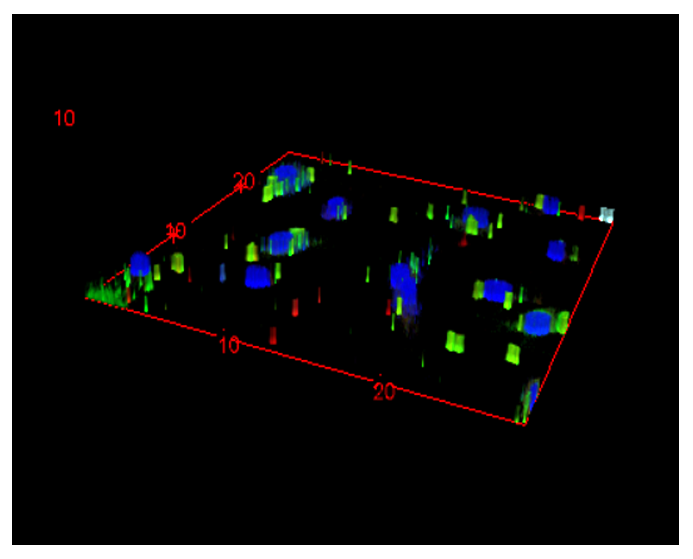


Supplementary Figure S2 3D view of the transfer of mitochondria within migrasomes (Red) to HUVECs (Green).
